# Supplementary material for: The effect of 3D stereopsis and hand-tool alignment on learning effectiveness and skill transfer of a VR-based simulator for dental training
Source: PLoS One. 2023 Oct 4;18(10):e0291389. doi: 10.1371/journal.pone.0291389 (PMC10550175; doi:10.1371/journal.pone.0291389)
Supplement: Attachment — Submitted filename: RebuttalLetter.docx [file pone.0291389.s002.docx]

Maximilian Kaluschke	17^th^ of August, 2023

Dear reviewers,

Thank you for your constructive comments. In the following I will explain how we tried to address each of your brought up points:

**Reviewer #1**

- Paragraph 2. Related work:
  In my humble opinion, some of the added information contain redundant information or justifications of your study which should be rephrased. For example:

Unnecessary justifications:
"... In contrast, we use an HMD for both conditions and disable stereopsis in the monoscopic 3D condition" (more appropriate in the discussion if you want to add this information to the manuscript)

- Thank you for pointing this out. We have removed the mentioned sentence all together, as we feel it didn’t fit well into the Discussion either.
- "To produce 2D images, the simulator was engineered to output a single image to both eyes, which is essentially the same way we achieved it" (reading this sentence it sounds too emotional regarding the comments of the reviewer)
  - Thank you for pointing this out. We removed the emotional sounding phrase.
- redundant information:
  "Their findings include that the participants from immersed groups either in preceptorship or training performed the anesthetic procedure faster and more accurately than those in the combined non-immersed groups. The authors conclude that the stereoscopic view from the HMD in immersed groups provided a better perception of depth when compared to the 2D monitor, makinginstrument navigation inside the mouth easier and leading to better performance results.
  The results showed that participants without immersive displays had less accurate needle insertion points, though needle injection angle and depth were not significantly different between the groups. The needle insertion point here needs to found without haptic feedback. As such it differs considerably from the root canal opening, since the bur can touch the tooth with drilling disabled for orientation with the help of haptic feedback". Please decide, which paragraph you prefer to summarize this paper.
  - Thank you for spotting this redundancy. We have removed the first paragraph.

**Reviewer #2**

- Comments have been addressed thoroughly. The paper is much clearer and a stronger work as a result of the authors revising the manuscript.
  - Thank you very much.

Best regards on behalf of

Maximilian Kaluschke, Myat Su Yin, Peter Haddawy, Siriwan Suebnukarn and Gabriel Zachmann
